# 非小细胞肺癌中PRL-3与RhoC的表达及相关性意义

**DOI:** 10.3779/j.issn.1009-3419.2010.06.006

**Published:** 2010-06-20

**Authors:** 平 张, 志培 张, 香敏 李, 杰 雷, 凯 苏, 小飞 李, 小平 王

**Affiliations:** 1 710038 西安，第四军医大学唐都医院胸腔外科 Thoracic Surgery, Tangdu Hospital, Fourth Military Medical University, Xi'an 710038, China; 2 810001 西宁，青海大学附属 医院肾内科 Department of Nephrology, Qinghai Uniersity Affiliated Hospital, Xining 810001, China

**Keywords:** 肺肿瘤, PRL-3, RhoC, 免疫组织化学, Lung neoplasms, PRL-3 protein, RhoC protein, Immunohistochemistry

## Abstract

**背景与目的:**

PRL-3是新近发现的酪氨酸蛋白磷酸酶，尚属于肝再生磷酸酶家族，具有促进肿瘤转移作用；RhoC属于小分子G蛋白超家族中的Rho亚家族，二者作用机制不清楚。本研究通过检测非小细胞肺癌（nonsmall cell lung cancer, NSCLC）中PRL-3和RhoC的表达，分析二者表达的相关性及在不同分组之间的差异，为进一步研究PRL-3在肿瘤发生发展中的作用机理提供实验依据。

**方法:**

采用免疫组化SP法检测PRL-3和RhoC在92例NSCLC中的表达，用统计学方法检验分析二者在不同分组间的表达差异性及二者的相关性。

**结果:**

在NSCLC中PRL-3和RhoC的表达阳性率分别为69.6%（64/92）、73.9%（68/92），二者的表达在不同的TNM分期、淋巴结及胸膜是否转移的分组之间差异有统计学意义（*P* < 0.01），同时二者的表达具有相关性（*r*=0.754, *P* < 0.001）。

**结论:**

在NSCLC中PRL-3和RhoC在TNM分期较高以及合并淋巴结、胸膜转移的分组中表达较高，同时二者的表达具有相关性，提示PRL-3可能通过RhoC及其下游因子促进癌细胞的远处转移。

## Abstract (en)

**Background and objective:**

Phosphatase of regenerating liver-3 (PRL-3) is a newly identified proteintyrosine phosphatase, which belongs to phosphatase of regenerating liver family, and plays a role in promoting tumor metastasis; Ras homologue C (RhoC) belongs to Rho subfamily of small-molecule G protein superfamily. However, the mechanisms of PRL-3 and RhoC are unknown. The aim of this study is to investigate the expressions of PRL-3 and RhoC proteins and their correlation to invasion and metastasis of non-small cell lung cancer (NSCLC), which may provide experiment evidence of the mechanism of PRL-3 in tumorigenesis and tumor-development.

**Methods:**

Immunohistochemical staining was used to detect the expressions of PRL-3 and RhoC in NSCLC in 92 cases, and statistical methods were used to analyse statistical significances of their expressions in different groups and their correlation.

**Results:**

The positive rates of PRL-3 and RhoC expressions in NSCLC were 69.6% (64/92) and 73.9% (68/92), respectively, and the expressions of PRL-3 and RhoC were closely correlated with TNM stage and lymphatic metastasis and pleural metastasis (*P* < 0.01), and they were correlated with each other (*r*=0.754, *P* < 0.001).

**Conclusion:**

The expressions of PRL-3 and RhoC are higher in the higher TNM stage and lymphatic metastasis and pleural metastasis cases, and closely correlate with each other in NSCLC, which suggests that PRL-3 and RhoC might be in the same signal pathway and PRL-3 might promote the distant metastasis of cancer cell by RhoC and downstream factors.

肺癌是一种严重威胁人类健康的恶性肿瘤，其发病率和死亡率呈上升趋势。我国数个大中城市中肺癌的发病率、死亡率已占男性恶性肿瘤的首位，女性恶性肿瘤的第二位^[1]^，对于这一疾病急需深入的研究。近年来的研究^[2-4]^发现，磷酸酶家族成员肝再生磷酸酶(PRL，包括PRL1-3共3种分子)参与多种类型肿瘤的发生与发展，其中PRL3在结直肠癌^[3, 5]^、卵巢癌^[6]^和胃癌^[7]^高表达中，在肿瘤细胞的增殖、粘附、迁移、侵袭以及转移中发挥了重要的正向调控作用。但有关PRL-3在肺癌不同病程中的表达情况及与肿瘤细胞侵袭、转移的关系鲜有报道。RhoC属于小分子G蛋白超家族中的Rho亚家族，是Rho信号转导通路的重要分子，许多研究表明RhoC在卵巢癌^[8]^、肝癌^[9]^、肺癌^[10]^等肿瘤中高表达并与肿瘤的浸润转移高度相关。Fiordalisi等^[11]^通过体外实验提示PRL-3可能是通过激活Rho信号转导通路来促进肿瘤的运动与转移。本研究应用免疫组化的方法检测PRL-3和RhoC在肺癌组织中的表达情况，探讨二者与肺癌临床病理特征的关系，分析其与肺浸润转移的关系，并探讨PRL-3与RhoC在非小细胞肺癌(non-small cell lung cancer, NSCLC)组织中的表达是否存在相关性。

## 1 材料与方法

### 1.1 材料与试剂

收集2008年6月-2009年6月第四军医大学唐都医院胸外科手术标本92例。其中鳞癌51例，腺癌41例；男性61例，女性31例；最大年龄72岁，最小年龄30岁，平均年龄57.83岁。将标本用10%中性甲醛溶液固定，脱水、透明、石蜡包埋。组织病理分类及分级由我院病理科医生确定，结合手术、影像学资料进行TNM分期。鼠抗人PRL-3单克隆抗体为Santa Cruz公司产品，兔抗鼠IgG/辣根酶标记二抗购自北京博奥森生物技术有限公司；兔抗人RhoC多克隆抗体为北京博奥森生物技术有限公司产品，羊抗兔IgG/辣根酶标记二抗和DAB显色试剂盒购自北京中杉金桥生物技术有限公司。

### 1.2 免疫组织化学染色

石蜡包埋的组织块经5 μm厚连续切片，分别做HE染色和免疫组织化学染色。免疫组化染色采用SP(streptavidin-perosidase)法。步骤如下：脱蜡至水、3 mol/L尿素溶液消化、3%过氧化氢溶液封闭、柠檬酸盐缓冲液微波修复抗原、自然冷却至室温、血清封闭、加一抗(稀释比例：抗PRL-3抗体为1:75，抗RhoC抗体为1:150)后4 ℃过夜、37 ℃复温、加二抗37 ℃反应、DAB显色、苏木素复染、盐酸酒精分化、脱水透明、中性树胶封片。用PBS液取代一抗做空白对照。

### 1.3 结果判断

PRL-3和RhoC免疫组化染色均位于细胞质和胞膜。每张组化结果随机选取5个不同的视野，分别计数阳性细胞数并计算其算术平均值。按照半定量积分方法判定，每例均随机观察5个高倍视野，判断结果：阳性细胞≤5%为0分，6%-25%为1分，26%-50%为2分，51%-75%为3分， > 75%为4分；阳性强度黄色为1分，棕黄色为2分，棕褐色为3分。将细胞阳性率与染色强度积分相乘，0分为阴性(-)，1分-4分为弱阳性(+)，5分-8分为中度阳性(++)，9分-12分为强阳性(+++)^[12]^。

### 1.4 统计学分析

应用SPSS 12.0软件进行统计学分析，临床病理特征分组统计采用*Mann-whitney U*检验，相关性分析采用*Spearman*检验，以*P* < 0.05为差异具有统计学意义。

## 2 结果

### 2.1 免疫组织化学染色表达定位

结果显示PRL-3和RhoC蛋白均定位于细胞质或细胞膜上，呈黄色或棕黄色颗粒(图 1)。

**1:**
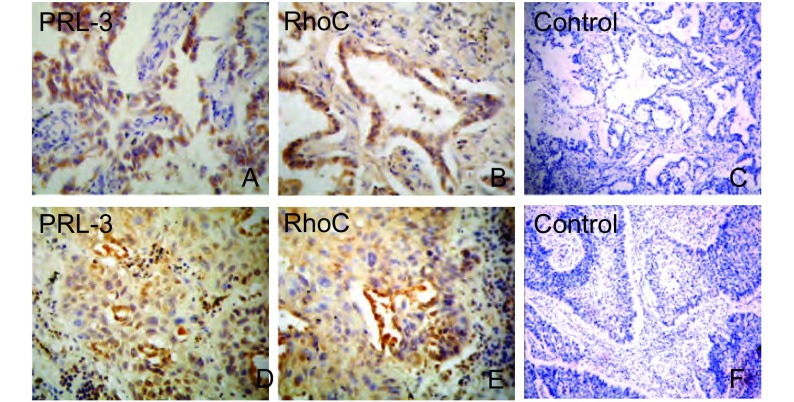
PRL-3、RhoC在肺癌组织中的表达。A、B、C:腺癌; D、E、F:鳞癌; A、D: PRL-3阳性表达; B、E:RhoC阳性表达; C、F空白对照（SP, A、B、D、E×400; C、F×200）。 Expressions of PRL-3, RhoC in lung cancer. A, B, C: lung adenocarcinome; D, E, F: squamous cell lung cancer; A, D: positive expression of PRL-3; B, E: positive expression of RhoC; C, F: blank control (SP, A, B, D, E×400; C, F×200).

### 2.2 临床病理特征分组统计结果

应用非参数检验中的*Mann-Whitney U*检验对PRL-3和RhoC蛋白在性别、年龄、病理类型、分化程度、TNM分期、是否伴淋巴结及胸膜转移7个分组之间进行统计分析，性别、年龄、病理类型、分化程度4个分组之间差异无统计学意义(*P* > 0.05)，而TNM分期、是否伴淋巴结及胸膜转移3个分组之间差异有统计学意义(*P* < 0.01)(表 1)。

**1:** PRL-3、RhoC在肺癌组织中的表达与临床病理特征之间的关系（*Mann-Whitney U*检验） The relationship between PRL-3, RhoC expressions in NSCLC and clinical pathological factors (*Mann-Whitney U* Test)

	PRL-3 expression							RhoC expression				
	n	-	+	++	+++	Statistics		-	+	++	+++	Statistics
Gender						*U*=807.500						*U*=872.500
Male	61	20	33	7	1	*P*=0.210		15	19	22	5	*P*=0.528
Female	31	8	15	7	1			9	11	8	3	
Age						*U*=1 012.500						*U*=1 040.500
≤60	47	14	24	7	2	*P*=0.699		10	19	14	4	*P*=0.889
> 60	45	14	24	7	0			14	11	16	4	
Histology						*U*=930.500						*U*=941.500
Squamous cancer	51	17	27	6	1	*P*=0.320		12	16	18	5	*P*=0.392
Adenocarcinoma	41	11	21	8	1			12	14	12	3	
Differentiation						*U*=960.000						*U*=823.000
Well and moderate	58	15	35	8	0	*P*=0.817		12	29	14	3	*P*=0.167
Poor	34	13	13	6	2			12	1	16	5	
TNM stage						*U*=653.000						*U*=545.000
Ⅰ-Ⅱ	46	22	19	5	0	*P*=0.001		21	15	6	4	*P* < 0.001
Ⅲ	46	6	29	9	2			3	15	24	4	
Lymph node metastasis						*U*=465.500						*U*=284.500
Positive	55	6	35	12	2	*P* < 0.001		2	19	26	8	*P* < 0.001
Negative	37	22	13	2	0			22	11	4	0	
Pleural metastasis						*U*= 601.500						*U*=465.500
Positive	41	5	24	10	2	*P* < 0.001		1	14	20	6	*P* < 0.001
Negative	51	23	24	4	0			23	16	10	2	

### 2.3 相关性分析

应用*Spearman*检验对PRL-3和RhoC蛋白在非小细胞肺癌中表达的强度进行相关性分析，结果显示二者的表达存在较强的相关性，有统计学意义(*r*=0.754, *P* < 0.001)(表 2)。

**2:** PRL-3与RhoC在非小细胞肺癌中表达相关性（*Spearman*检验） The correlation of PRL-3 expression with RhoC expression in NSCLC (*Spearman* test)

RhoC	PRL-3				Total	Statistics
	-	+	++	+++		
-	22	4	0	0	24	
+	6	20	4	0	30	
++	0	25	4	0	30	
+++	0	1	5	2	8	*r*=0.754
Total	28	49	13	2	92	*P* < 0.001

## 3 讨论

人类*PRL-3*基因位于染色体8q24.3，编码173个氨基酸，大约75%的氨基酸序列与其家族中其他两个成员PRL-1、PRL-2相同，其分子质量约为20 kDa，是非典型磷酸酪氨酸蛋白磷酸脂酶。在人类正常组织中，PRL-3主要在心肌、平滑肌和骨骼肌中表达^[13]^。Saha等^[3]^应用基因表达系列分析(SAGE)发现*PRL-3*基因在结直肠癌转移组织中过度表达，进一步研究发现PRL-3在正常结、直肠粘膜和无转移原位癌中低度表达，在有转移的原发癌中则中度表达，而且在原发癌切除术后出现肝肺等远处转移的机率随原发癌组织中PRL-3高表达而增加。目前，对PRL-3在NSCLC组织的表达与临床病理特征之间的关系鲜有报道。

本实验通过分析PRL-3在不同的性别、年龄、病理类型、分化程度、TNM分期、淋巴结转移、胸膜转移分组的表达情况，发现PRL-3的表达在不同的性别、年龄、病理类型、分化程度无明显差别，而随着TNM分期的增高、合并淋巴结转移及胸膜转移，PRL-3的表达明显增高，这一现象提示PRL-3可能有促进NSCLC转移的功能。

*RhoC*基因定位于1p13-p21，RhoC蛋白含193个氨基酸，其分子量在20 kDa-30 kDa之间，属于Ras超家族中的小GTP结合蛋白，在真核细胞中作为分子开关控制着众多信号转导途径^[14]^。RhoC蛋白在细胞的信号转导通路中作为信号转换器，调控各种细胞骨架运动、细胞形态建成等功能^[15]^，通过对细胞骨架蛋白的调节加快细胞迁移^[16, 17]^。

本实验通过分析RhoC在不同的性别、年龄、病理类型、分化程度、TNM分期、淋巴结转移、胸膜转移分组的表达情况，发现RhoC的表达在不同的性别、年龄、病理类型、分化程度无明显差别，而随着TNM分期的增高、合并淋巴结转移及胸膜转移，RhoC的表达明显增高，这一现象提示RhoC可能有促进NSCLC转移的功能。

本实验采用免疫组化SP法检测PRL-3和RhoC在92例NSCLC的表达，二者的表达阳性率分别为69.6%(64/92)、73.9%(68/92)，表达的强弱在不同的TNM分期、淋巴结及胸膜是否转移的分组之间具有显著的差异性，同时二者的表达强弱具有较强的相关性。从统计结果可以得出：在转移能力较强的NSCLC中PRL-3和RhoC蛋白的表达较高，二者可能具有促进癌细胞远处转移的生物学功能，这一结论证实了现阶段对这两个蛋白的功能的认识。目前RhoC蛋白比较明确的功能是引起F-肌动蛋白的重组和动态变化^[18]^，但是在该通路中RhoC蛋白活化及其信号传递机制还不是很清楚，结合本实验结果以及Fiordalisi等^[11]^、Ming等^[19]^的实验结果，PRL-3可能对RhoC蛋白的表达有调节功能，可能通过RhoC蛋白促进NSCLC远处转移，但PRL-3作为磷酸酶直接的作用是使底物去磷酸化，而RhoC蛋白只有在结合GTP(即磷酸化状态)时才具有生物学活性，可以推论出PRL-3不是直接作用于RhoC蛋白来发挥其生物学作用，而有可能是通过一些未知的分子来调节RhoC蛋白，再通过其下游因子促进癌细胞的远处转移。目前已知磷酸化与脱磷酸化修饰在体内各种代谢的调节中及受体功能调节上起重要作用，PRL-3作为一种磷酸酶可能通过其脱磷酸化功能参与体内某些代谢的调节及某些受体功能调节，从而在肿瘤的发生发展中发挥重要作用。PRL-3促进肿瘤细胞远处转移的作用机制仍不清楚，还需要进一步的实验来阐明，同时RhoC蛋白对PRL-3是否具有反馈作用也需要更深入的研究。
